# Impact of *CHRNA5* polymorphisms on the risk of schizophrenia in the Chinese Han population

**DOI:** 10.1002/mgg3.869

**Published:** 2019-07-24

**Authors:** Dafei Zhan, Qiankun Yao, Shaojian Fu, Xianglai Liu, Jun Zhou, Daqiang Chen, Chuanlong Yu

**Affiliations:** ^1^ Division of Prevention Hainan Provincial Anning Hospital Haikou China

**Keywords:** *CHRNA5*, Neuronal nicotinic acetylcholine receptors, Schizophrenia, Single nucleotide polymorphism

## Abstract

**Background:**

Schizophrenia is a complex mental disease whose cause is still unknown. Neuronal nicotinic acetylcholine receptors (nAChRs) have been implicated in various neurological disorders, including schizophrenia. The previous reports have shown that *CHRNA* polymorphisms were involved in schizophrenia. This study is to explore the potential association between *CHRNA5* (OMIM#118505) polymorphisms and schizophrenia susceptibility in a Chinese population.

**Methods and Results:**

A case–control study was conducted with 384 schizophrenia patients and 687 controls. We genotyped eight single nucleotide polymorphisms (SNPs) distributed in *CHRNA5*. Regulome DB, HaploReg, and GTEx databases were used to calculate possible functional effects of the polymorphisms. The χ^2^ test, genetic model analysis, and haplotype analysis were involved in assessing genetic association between variants and schizophrenia risk. The results exhibited that rs17486278 (NC_000015.10:g.78575140A>C) was associated with a decreased risk of schizophrenia on the basis of the recessive model (adjusted OR = 0.37, 95%CI: 0.15–0.93) in females. Moreover, we found that the four variants rs588765, rs6495306, rs680244, rs692780 were extremely significant after being stratified by ≥45 years.

**Conclusions:**

Overall, our findings supported that the potential association existed between *CHRNA5* polymorphisms and schizophrenia susceptibility in a Chinese population. But, large sample validation is needed to enhance the accuracy of our results.

## 1 INTRODUCTION

Schizophrenia, a serious mental illness, is characterized by the disharmony between mental activity and the realistic environment, the disharmony and disintegration of cognition, emotion, volition, and so forth. It has shortened the lifespan and afflicts about 1% of the population all over the world (Leucht, Burkard, Henderson, Maj, & Sartorius, 2007). Symptoms typically occur in late adolescence or early adulthood. Not surprisingly, the financial burden of this disease is considerable. But so far, the etiology of schizophrenia remains unknown and is still in the exploratory stage. There was no theory that could perfectly explain the pathogenesis of schizophrenia. It is generally believed that the occurrence of schizophrenia is mainly influenced by environment and genetic background, among which genetic factors account for 80%. Family and twin studies of schizophrenia found that the prevalence of relatives was significantly higher than that of the general population. The heritability of schizophrenia and bipolar disorder is between 70% and 80%, with major depressive disorder up to 40% (Uher, 2014).

Over the past few decades, a link between genetic polymorphisms and schizophrenia has been proposed. Numerous researchers are trying to understand the association of genetic polymorphism with schizophrenia to understand the etiology of schizophrenia. Recently there has been a greater focus on candidate gene *CHRNA5* (OMIM#118505) encoding the α5 subunit of nicotinic acetylcholine receptors (nAChRs) in the studies of schizophrenia pathophysiology. *CHRNA5* is located in 15q25.1 and belongs to the superfamily of ligand‐gated ion channels that mediated fast signal transmission at synapses. The present study follows up on data reported by some researchers by examining *CHRNA5* polymorphisms and lung cancer risk in the population (Huang et al., 2015; Shen et al., 2013; Xu et al., 2015), suggesting that genetic variation in *CHRNA5* may affect susceptibility to lung cancer among smokers. But, few studies have been reported in the correlation between *CHRNA5* polymorphisms and schizophrenia.

To examine whether *CHRNA5* may also contribute to schizophrenia in a Chinese population, we selected eight variants in *CHRNA5* to perform a case–control study. Herein, the results reported may help in future studies of schizophrenia and contribute to easing the burden of this disease on individuals, families, and society.

## 2 MATERIALS AND METHODS

### 2.1 Ethical compliance

All participants have been informed both verbally and in writing of the procedures and purpose of this study and they signed informed consent documents. This study protocol was approved by the Clinical Research Ethics Committees of Psychiatric Hospital of Xi'an. All the subsequent research analyses were carried out in accordance with Department of Health and Human Services (DHHS) regulations for human research subject protection.

### 2.2 Study subjects

We recruited 384 schizophrenia patients from Psychiatric Hospital of Xi'an, Shaanxi province and 687 volunteers were considered as controls to conduct a case–control study. On the basis of DSM‐IV (Diagnostic and Statistical Manual of Mental Disorders, the fourth version), all patients were diagnosed and pathologically confirmed by the experienced senior psychiatrists to suffer from schizophrenia. The patients had no history of other related diseases, including cancer, nephropathy, and so on. Also, there were no sex, age, and/or stage restrictions for cases and none of the healthy control subjects had any mental illness. Besides, all the participants were genetically unrelated ethnic Han Chinese.

### 2.3 SNP selection and genotyping

On the basis of the dbSNP database, we randomly selected eight candidate polymorphisms (rs667282, rs16969948, rs588765, rs6495306, rs17486278, rs680244, rs569207, and rs692780) in *CHRNA5* (its GenBank reference is NC_000015.10). Each SNP had minor allele frequency (MAF)> 5% in the global population from 1,000 Genome Projects (http://www.internationalgenome.org/). Then Regulome DB (http://www.regulomedb.org/) and HaploReg were utilized to predict the function of SNPs. We used the Genotype‐Tissue Expression (GTEx) projects (https://gtexportal.org/home/) expression quantitative trait loci (eQTL) variants to assess the effects of schizophrenia‐associated SNPs on gene expression. Genomic DNA was extracted from blood samples using the GoldMag‐Mini Whole Blood Genomic DNA Purification Kit (GoldMag Ltd. Xi'an City, Shaanxi, China). NanoDrop2000 (Thermo Scientific, Waltham, Massachusetts, USA) was used to check the quantification of the extracted DNA at a wavelength value of A260 nm. We used the Agena MassARRAY Assay Design 3.0 Software (San Diego, CA) to design Multiplexed SNP MassEXTEND assays and genotyped the variants using the MassARRAY iPLEX (Agena Bioscience) platform using the matrix‐assisted laser desorption ionization‐time of flight (MALDITOF). Data management and analysis was conducted by the Agena Typer 4.0 software (San Diego, CA).

### 2.4 Statistical analyses

Statistical analysis was performed by SPSS 20.0 software (SPSS Inc.). Fisher's exact test was used to analyze the genotype frequencies for each SNP to evaluate if they deviated from Hardy–Weinberg equilibrium (HWE) in this study. Pearson's χ^2^ test was used to calculate the allele and genotype frequencies of each SNP between patients with schizophrenia and controls. Genetic models were generated using SNPStats (https://www.snpstats.net/start.htm?q=snpstats/start.htm) software to estimate the relationship between each SNP and schizophrenia risk. The odds ratio (OR) and 95% confidence interval (CI) were calculated by logistic regression analysis adjusted for age and gender. Eventually, we estimated the linkage disequilibrium (LD) on the Haploview software (version 4.2)(Barrett, Fry, Maller, & Daly, 2005). Power and Sample Size (PS) Calculation software (http://biostat.mc.vanderbilt.edu/wiki/Main/PowerSampleSize#Downloading_and_Installing_the_PS_Software) was utilized to calculate the power of the significant difference(Dupont and Plummer 1998). All *p* values were two‐sided, and *p* < .05 was considered as statistically significant site.

## 3 RESULTS

### 3.1 Characteristics of case and control

Of the 1,071 samples (588 men and 483 women), 384 were classified as schizophrenia cases (201 men and 183 women), and 687 were classified as controls (300 men and 387 women) in the study. The average age of the initially diagnosed schizophrenia and normal people was 36.58 ± 13.733 years and 48.56 ± 9.559 years, respectively.

### 3.2 The association between *CHRNA5* SNPs and schizophrenia risk

We successfully genotyped eight SNPs listed in Tables 1 and 2. The Regulome DB scores and HaploReg were the function of the selected SNPs shown in Table 1. We analyzed the data, including four gene models (codominant, dominant, recessive, and additive models) to explore the correlation between SNPs and schizophrenia by logistic regression analysis with adjustment for age and gender. Eight variants (rs667282, rs16969948, rs588765, rs6495306, rs17486278, rs680244, rs569207, and rs692780) in the *CHRNA5* were found to be risk factors, but, there were no significant differences.

**Table 1:** In silico analysis for SNPs function annotation

SNP	Chr	Gene	Allele	RegulomeDB Score	HaploReg
rs667282	15q25.1	*CHRNA5*	C < T	5	Proteins bound, Motifs changed, Selected eQTL hits
rs16969948	15q25.1	*CHRNA5*	G < A	5	Motifs changed
rs588765	15q25.1	*CHRNA5*	T < C	No data	Selected eQTL hits
rs6495306	15q25.1	*CHRNA5*	G < A	1f	DNAse, Proteins bound, Motifs changed, Selected eQTL hits
rs17486278	15q25.1	*CHRNA5*	C < A	2b	Proteins bound, Motifs changed
rs680244	15q25.1	*CHRNA5*	T < C	No data	Selected eQTL hits
rs569207	15q25.1	*CHRNA5*	T < C	No data	Motifs changed, Selected eQTL hits
rs692780	15q25.1	*CHRNA5*	C < G	No data	Motifs changed, Selected eQTL hits

Note

1f indicates that the variant is likely to affect binding and linked to expression of a gene target.

2b indicates that the variant is likely to affect binding.

5 indicates that the variant has minimal binding evidence.

The GenBank reference of *CHRNA5*: NC_000015.10.

**Table 2:** Basic information and allele frequencies of the SNPs in *CHRNA5*

SNP	Chromosome	Position	Alleles A < B	Role	Minor Allele Frequency (A)		HWE *p*	OR (95%CI)	*p* *
					Case	Control			
rs667282	chr15	78,863,472	C < T	Intron	0.466	0.461	1.000	1.02 (0.85–1.22)	.834
rs16969948	chr15	78,864,786	G < A	Intron	0.050	0.055	1.000	0.89 (0.60–1.33)	.573
rs588765	chr15	78,865,425	T < C	Intron	0.241	0.207	.907	1.22 (0.99–1.50)	.067
rs6495306	chr15	78,865,893	G < A	Intron	0.240	0.207	.907	1.21 (0.98–1.49)	.080
rs17486278	chr15	78,867,482	C < A	Intron	0.247	0.277	1.000	0.86 (0.70–1.05)	.834
rs680244	chr15	78,871,288	T < C	Intron	0.288	0.265	.492	1.12 (0.92–1.37)	.573
rs569207	chr15	78,873,119	T < C	Intron(boundary)	0.464	0.461	.939	1.01 (0.85–1.21)	.067
rs692780	chr15	78,876,505	C < G	Intron	0.237	0.214	.734	1.14 (0.92–1.41)	.080

Note

Abbreviations: SNP: Single nucleotide polymorphism, OR: odd ratio, 95% CI: 95% confidence interval, HWE: Hardy–Weinberg equilibrium.

The GenBank reference of *CHRNA5*: NC_000015.10.

* *p*‐values obtained from Pearson χ^2^ test.

### 3.3 Stratification analysis by gender and age

We reanalyzed the case–control association study by gender stratifying. In Table 3, after the adjustment for age, the frequency of the homozygous “C/C” genotype of rs17486278 (HGVS: NM_000745.3:g.78575140A>C) differed significantly between schizophrenia patients and controls (4.4% vs. 7.7%) in females. The variant was associated with a decreased risk of schizophrenia in the recessive model (adjusted OR = 0.37, 95%CI: 0.15–0.93) with power values of 0.982 in females.

**Table 3:** Rs17486278 associated with the susceptibility of schizophrenia in females and males

Model	Genotype	Control(%)	Case(%)	Adjustment With Age in females		Adjustment With Age in males	
				OR (95%CI)	*p* *	OR (95%CI)	*p* *
Codominant	A/A	163 (54.3)	107 (59.4)	1.00		1.00	
	C/A	114 (38)	65 (36.1)	0.95 (0.61–1.48)	.081	0.79 (0.53–1.19)	.530
	C/C	23 (7.7)	8 (4.4)	0.36 (0.14–0.92)		0.94 (0.46–1.92)	
Dominant	A/A	163 (54.3)	107 (59.4)	1.00	.360	1.00	.310
	C/A‐C/C	137 (45.7)	73 (40.6)	0.82 (0.54–1.25)		0.82 (0.56–1.20)	
Recessive	A/A‐C/A	277 (92.3)	172 (95.6)	1.00	**.026**	1.00	.920
	C/C	23 (7.7)	8 (4.4)	**0.37 (0.15–0.93)**		1.03 (0.52–2.08)	
Log‐additive	–	–	–	0.75 (0.54–1.06)	1.000	0.89 (0.66–1.20)	.450

Note

Abbreviations: OR: odds ratio, 95% CI, 95% confidence interval.

The GenBank reference of *CHRNA5*: NC_000015.10.

* *p*‐values were calculated by logistic regression analysis with adjustment by age. Bold type indicates that the locus has statistically significant (*p* < .05).

Moreover, we found that the four variants rs588765, rs6495306, rs680244, rs692780 were significantly linked to the risk of schizophrenia after adjusted by ≥45 years (Table 4), but none of the sites exhibilited the association with schizophrenia risk with adjustment by <45 years. The four SNPs were noteworthy before and after the adjustment. The rs588765 (HGVS: NM_000745.3:g.78573083T>C) and rs680244 (HGVS: NM_000745.3:g.78578946T>C) genotype for “C/T” subjects were linked to an increased risk of schizophrenia based on the results of the codominant model (adjusted OR = 2.12, 95%CI: 1.36–3.30, *p* = .003; adjusted OR = 1.70, 95%CI: 1.09–2.67, *p* = .027, respectively) with power values of 0.879 and 0.646, respectively. In addition, allele “T” of the rs588765 and rs680244 significantly increased the risk of schizophrenia in the dominant model (adjusted OR = 2.12, 95%CI: 1.38–3.25, *p* = 6e‐04; adjusted OR = 1.77, 95%CI: 1.15–2.71, *p* = .009, respectively) with power values of 0.879 and 0.710, respectively. In the log‐additive model, the obtained results were basically consistent.

**Table 4:** Significant variants in *CHRNA5* associated with schizophrenia susceptibility after being stratified by ≥ 45 years

	SNP	Model	Genotype	Control(%)	Case(%)	Without Adjustment		Adjustment With Gender and Age	
						OR (95%CI)	*p* a	OR (95%CI)	*p* b
*CHRNA5*	rs588765 (call rate 100%)	Codominant	C/C	290 (64.9)	50 (46.3)	1.00	**.002**	1.00	**.003**
			C/T	136 (30.4)	50 (46.3)	**2.13 (1.37–3.32)**		**2.12 (1.36–3.30)**	
			T/T	21 (4.7)	8 (7.4)	2.21 (0.93–5.26)		2.13 (0.89–5.09)	
		Dominant	C/C	290 (64.9)	50 (46.3)	1.00	**4e−04**	1.00	**6e−04**
			C/T‐T/T	157 (35.1)	58 (53.7)	**2.14 (1.40–3.28)**		**2.12 (1.38–3.25)**	
		Recessive	C/C‐C/T	426 (95.3)	100 (92.6)	1.00	.280	1.00	.320
			T/T	21 (4.7)	8 (7.4)	1.62 (0.70–3.77)		1.56 (0.67–3.64)	
		Log‐additive	–	–	–	**1.76 (1.26–2.45)**	**.001**	**1.73 (1.24–2.43)**	**.002**
	rs6495306 (call rate 100%)	Codominant	A/A	290 (64.9)	50 (46.3)	1.00	**.002**	1.00	**.003**
			A/G	136 (30.4)	50 (46.3)	**2.13 (1.37–3.32)**		**2.12 (1.36–3.30)**	
			G/G	21 (4.7)	8 (7.4)	2.21 (0.93–5.26)		2.13 (0.89–5.09)	
		Dominant	A/A	290 (64.9)	50 (46.3)	1.00	**4e−04**	1.00	**6e−04**
			A/G‐G/G	157 (35.1)	58 (53.7)	**2.14 (1.40–3.28)**		**2.12 (1.38–3.25)**	
		Recessive	A/A‐A/G	426 (95.3)	100 (92.6)	1.00	.280	1.00	.320
			G/G	21 (4.7)	8 (7.4)	1.62 (0.70–3.77)		1.56 (0.67–3.64)	
		Log‐additive	–	–	–	**1.76 (1.26–2.45)**	**.001**	**1.73 (1.24–2.43)**	**.002**
	rs680244 (call rate 99.82%)	Codominant	C/C	246 (55.2)	44 (40.7)	1.00	**.023**	1.00	**.027**
			C/T	171 (38.3)	53 (49.1)	**1.73 (1.11–2.70)**		**1.70 (1.09–2.67)**	
			T/T	29 (6.5)	11 (10.2)	2.12 (0.99–4.56)		2.14 (0.99–4.62)	
		Dominant	C/C	246 (55.2)	44 (40.7)	1.00	**.007**	1.00	**.009**
			C/T‐T/T	200 (44.8)	64 (59.3)	**1.79 (1.17–2.74)**		**1.77 (1.15–2.71)**	
		Recessive	C/C‐C/T	417 (93.5)	97 (89.8)	1.00	.200	1.00	.190
			T/T	29 (6.5)	11 (10.2)	1.63 (0.79–3.38)		1.66 (0.80–3.45)	
		Log‐additive	–	–	–	**1.56 (1.13–2.15)**	**.008**	**1.55 (1.12–2.15)**	**.009**
	rs692780 (call rate 100%)	Codominant	G/G	286 (64)	50 (46.3)	1.00	**.004**	1.00	**.005**
			C/G	139 (31.1)	51 (47.2)	**2.10 (1.35–3.26)**		**2.07 (1.33–3.22)**	
			C/C	22 (4.9)	7 (6.5)	1.82 (0.74–4.49)		1.76 (0.71–4.35)	
		Dominant	G/G	286 (64)	50 (46.3)	1.00	**8e−04**	1.00	**.001**
			C/G‐C/C	161 (36)	58 (53.7)	**2.06 (1.35–3.15)**		**2.02 (1.32–3.10)**	
		Recessive	G/G‐C/G	425 (95.1)	101 (93.5)	1.00	.520	1.00	.570
			C/C	22 (4.9)	7 (6.5)	1.34 (0.56–3.22)		1.30 (0.54–3.13)	
		Log‐additive	–	–	–	**1.67 (1.19–2.33)**	**.003**	**1.64 (1.17–2.30)**	**.005**

Note

Abbreviations: SNP: Single nucleotide polymorphism, OR: odds ratio, 95% CI: 95% confidence interval.

The GenBank reference of *CHRNA5*: NC_000015.10.

Bold type indicates that the locus has statistically significant (*p* < .05).

a *p*‐values were calculated by logistic regression analysis.

b *p*‐values were calculated by logistic regression analysis with adjustment by gender and age.

The heterozygous genotype of rs6495306 (HGVS: NM_000745.3:g.78573551G>A) and rs692780 (HGVS: NM_000745.3:g.78584163C>G) presented an increased contribution to the risk of schizophrenia in the codominant model (adjusted OR = 2.12, 95%CI: 1.36–3.30, *p* = .003; adjusted OR = 2.07, 95%CI: 1.33–3.22, *p* = .005) with power values of 0.879 and 0.862, respectively. An association between allele “G” of rs6495306, allele “C” of rs692780, and an increased risk of schizophrenia were observed based on the dominant model (adjusted OR = 2.12, 95%CI: 1.38–3.25, *p* = 6e−04; adjusted OR = 2.02, 95%CI: 1.32–3.10, *p* = .001; respectively) with power values of 0.879 and 0.838, respectively. The two sites in the log‐additive model showed similar results. In addition, GTEx results (Table S1) showed that the statistically significant variants (rs17486278, rs588765, rs6495306, rs680244, rs692780) were associated with *CHRNA5* gene expression in the most relevant tissue (brain).

### 3.4 Linkage disequilibrium analysis and haplotypes

**Figure 1:**
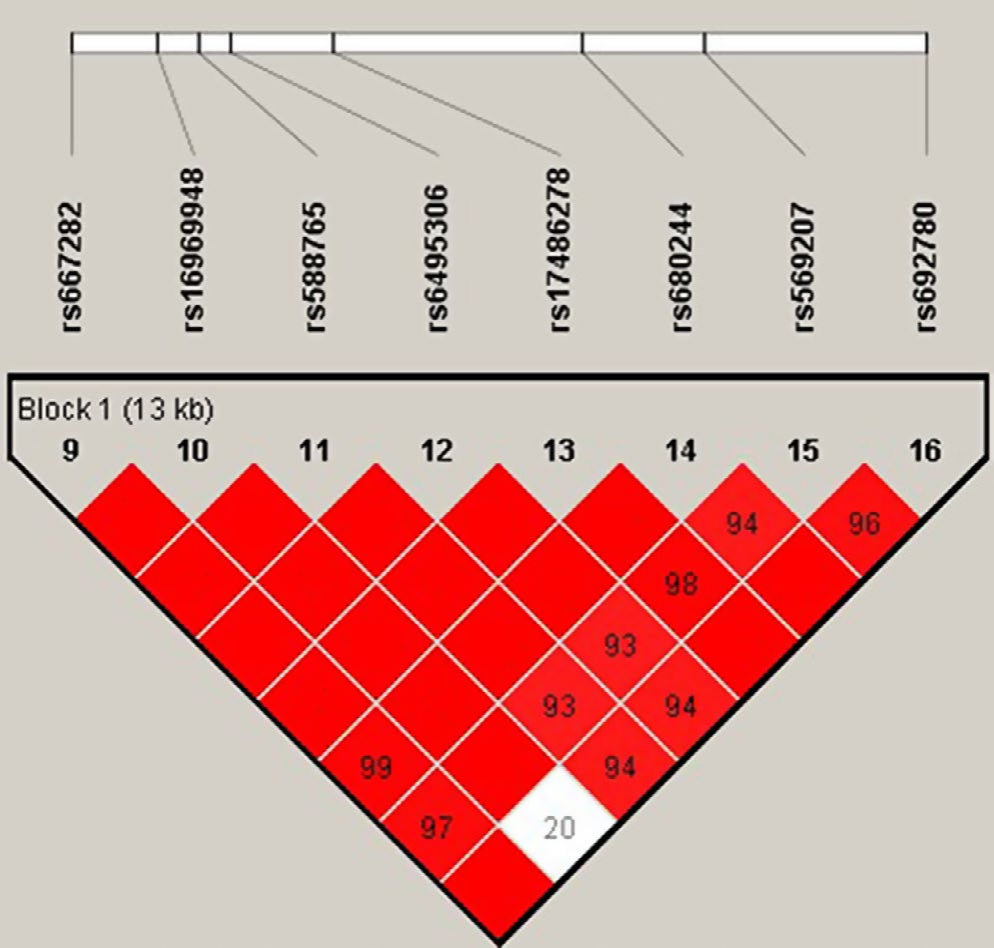
Linkage disequilibrium (LD) analysis of six SNPs in *CHRNA5*. Standard color schemes indicate different levels of LD. Dark red: LOD > 2, D’ = 1. LOD: logarithm of odds, SNP: single nucleotide polymorphism

**Figure 2:**
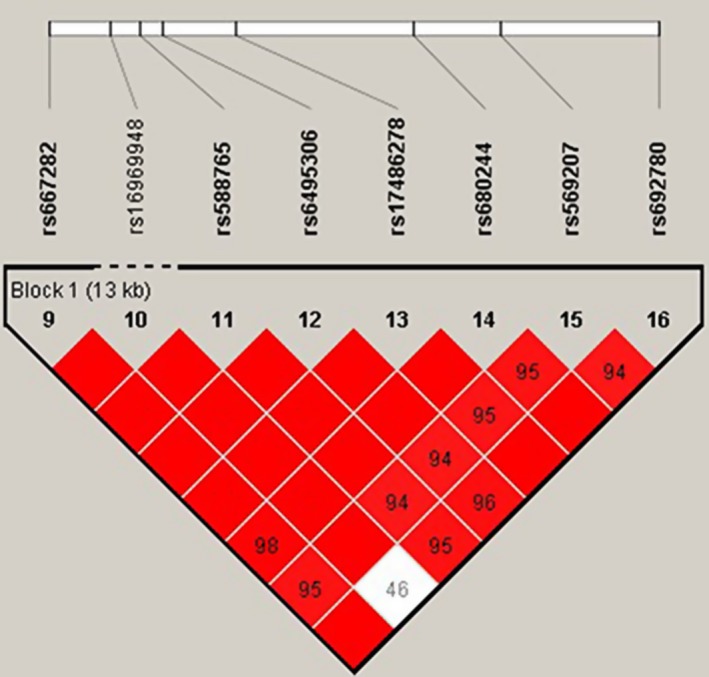
Linkage disequilibrium (LD) analysis of six SNPs in *CHRNA5* after adjusted by ≥ 45 years. Standard color schemes indicate different levels of LD. Dark red: LOD > 2, D’ = 1. LOD: logarithm of odds; SNP: single nucleotide polymorphism

Then we used linkage disequilibrium analysis to detect the correlation between *CHRNA5* SNPs, shown in Figures 1 and 2. In Figure 2, aside from rs16969948 (HGVS: NM_000745.3: g.78572444A>G), the significant association among other variants still existed in females. However, after stratifying analysis by ≥45 years, there existed higher linkage among eight *CHRNA5* SNPs (Figure 2).

Eventually, haplotype analysis was done in eight SNPs (rs667282, rs16969948, rs588765, rs6495306, rs17486278, rs680244, rs569207, and rs692780) and displayed five haplotypes in our controls and patients (Table 5). We found that the haplotype “TATGATCC” was linked to a higher risk of schizophrenia without or with adjustment by age and gender.

**Table 5:** Haplotype analysis between *CHRNA5* haplotypes and schizophrenia risk

	Haplotype								Freq	Without adjustment		Adjustment with gender and Age	
	rs667282	rs16969948	rs588765	rs6495306	rs17486278	rs680244	rs569207	rs692780		OR(95%CI)	*p* a	OR(95%CI)	*p* b
Block	C	A	C	A	A	C	T	G	0.457	1.00	–	1.00	–
	T	A	C	A	C	C	C	G	0.260	0.94 (0.63–1.42)	.780	0.95 (0.64–1.43)	.820
	T	A	T	G	A	T	C	C	0.205	**1.63 (1.12–2.39)**	**.012**	**1.62 (1.11–2.37)**	**.014**
	T	G	C	A	A	T	C	G	0.040	0.59 (0.21–1.62)	.300	0.61 (0.22–1.68)	.340
	T	G	C	A	A	T	C	C	0.014	2.02 (0.62–6.59)	.250	2.05 (0.63–6.69)	.240

Note

Abbreviations: Freq: frequence, OR: odds ratio, 95% CI: 95% confidence interval.

The GenBank reference of *CHRNA5*: NC_000015.10.

a *p*‐values were calculated by Wald test without adjustment.

b *p*‐values were calculated by Wald test adjusted by gender and age.

## 4 DISCUSSION

In our study, we investigated the correlations between eight *CHRNA5* SNPs and schizophrenia risk. Rs17486278 showed a significant association with decreased susceptibility of schizophrenia in females and males. After being stratified by ≥45 years, *CHRNA5* variants (rs588765, rs6495306, rs680244 and rs692780) were associated with an increased risk of schizophrenia. So, we deduced that the polymorphisms of *CHRNA5* may influence susceptibility to lung cancer among the Chinese populations.

The current antipsychotics mainly alleviate neurotransmitter imbalance, but most patients still have relapse in the current treatment plan (Leucht et al., 2013, 2009). For decades, pathophysiological studies related to schizophrenia were greatly focused on interfering with dopaminergic and glutamatergic neurotransmission to achieve the treatment of schizophrenia. *CHRNA5* is a member of nAChRs typically expressed in the nervous system and involved in various functional processes, including cognition, learning, memory, and so forth. Moreover, alterations in their expression and/or activity have been implicated in various neurological disorders, such as Alzheimer's disease (AD) (Dineley et al., 2001), Parkinson's disease (Xie, Gao, Xu, & Meng, 2014), and schizophrenia (Freedman, Adams, & Leonard, 2000). In general, nAChRs form a family composed of ligand‐gated cationic channels, activated by the endogenous neurotransmitter acetylcholine (ACh), carbachol, and nicotine to promote tumor development (Hung et al., 2008). In knockout mouse studies of *Chrna5,* mRNA expression in habenular and ventral tegmental area, mice with a null mutation for Chrna5 significantly increased nicotine intake by modulating the sensitivity of dopaminergic neurons (Fowler, Lu, Johnson, Marks, & Kenny, 2011; Morel et al., 2014). In the medial habenula, *Chrna5* overexpression can reduce nicotine consumption to wild‐type levels (Fowler et al., 2011), illustrating that *CHRNA5* mediates negative reward signaling through the habenulo‐interpenduncular pathway in the habenula.

Notably, polymorphisms located in a cluster of genes coding for the subunits α5, α3, and β4 of nAChR were related to various health problems (Bierut, 2010). *CHRNA5* rs16969968 was reported to interact with a splicing SNP in the *dopamine D2 receptor gene* (*DRD2*) involved in addiction (Moyer et al., 2011) to influence multiple aspects of prefrontal cortex physiology and behavior during working memory (Di Giorgio et al., 2014), suggesting that *CHRNA5* had a pervasive functional profile in brain regions central to cognition. Also, the previously reported studies have demonstrated that *CHRNA* variants have been linked to increased risk of lung cancer amongst Han individuals (He et al., 2014; Le Marchand et al., 2008; Niu et al., 2010; Thorgeirsson et al., 2008; Zhou et al., 2015). But, *CHRNA3* (OMIM#118503) polymorphisms were found to contribute to an increased risk of lung cancer in the Han individuals who smoke (Zhou et al., 2015), however, *CHRNA3* variant (rs8042374, NM_000743.4:g.78615690A>G) was linked to a greater risk of lung adenocarcinoma in female nonsmokers (He et al., 2014). Thus, the effect of smoking on the risk of lung diseases is not essential.

In our study, our case–control data provided statistical evidence for a strong association between *CHRNA5* (rs17486278, rs588765, rs6495306, rs680244, rs692780) and schizophrenia risk. Among them, rs17486278 was found to be an increased variant associated with lung cancer risk in African Americans and European populations (Broderick et al., 2009; Hansen et al., 2010). In a never‐smoking Chinese population study, the association between rs17486278 in gene cluster CHRNA5‐CHRNA3‐CHRNB4 and nonsmall cell lung cancer (NSCLC) was not significant (Li, Bao, Xu, Bao, & Zhang, 2012). While we evaluated the relationship between rs17486278 and lung cancer risk, it was consistent with the previous results of the Chinese population (Huang et al., 2015). The main reason for this difference should be racial differences. The SNP had no significance in males, but was statistically significant in females, so, the result of rs17486278 is different for gender differences.

Moreover, two variants (rs588765 and rs680244) have been reported to be correlated with an increased risk of lung cancer (Huang et al., 2015). Rs588765 and rs680244 were also associated with neuroticism (Criado, Gizer, Edenberg, & Ehlers, 2014). In this study, we firstly provide a new evidence that rs588765 and rs680244 appeared to be related to an increased schizophrenia risk in Shaanxi Han population study. But, having additional large sample series is necessary for further verification results to become more credible.

## 5 CONCLUSIONS

In conclusion, our results obtained from a cohort of Chinese schizophrenia patients illustrated that *CHRNA5* SNPs (rs17486278, rs588765, rs6495306, rs680244, rs692780) were significantly associated with schizophrenia risk. Hence, it may potentially serve as a clinically prediagnostic marker.

## CONFLICT OF INTERESTS

The authors declare no competing interests.

## References

[mgg3869-bib-0001] Barrett, J. C. , Fry, B. , Maller, J. , & Daly, M. J. (2005). Haploview: Analysis and visualization of LD and haplotype maps. Bioinformatics, 21, 263–265. 10.1093/bioinformatics/bth457 15297300

[mgg3869-bib-0002] Bierut, L. J. (2010). Convergence of genetic findings for nicotine dependence and smoking related diseases with chromosome 15q24‐25. Trends in Pharmacological Sciences, 31, 46–51. 10.1016/j.tips.2009.10.004 19896728 PMC2993565

[mgg3869-bib-0003] Broderick, P. , Wang, Y. , Vijayakrishnan, J. , Matakidou, A. , Spitz, M. R. , Eisen, T. , … Houlston, R. S. (2009). Deciphering the impact of common genetic variation on lung cancer risk: A genome‐wide association study. Cancer Research, 69, 6633–6641. 10.1158/0008-5472.CAN-09-0680 19654303 PMC2754318

[mgg3869-bib-0004] Criado, J. R. , Gizer, I. R. , Edenberg, H. J. , & Ehlers, C. L. (2014). CHRNA5 and CHRNA3 variants and level of neuroticism in young adult Mexican American men and women. Twin Research and Human Genetics, 17, 80–88. 24588897 10.1017/thg.2014.11 PMC4034688

[mgg3869-bib-0005] Di Giorgio, A. , Smith, R. M. , Fazio, L. , D'Ambrosio, E. , Gelao, B. , Tomasicchio, A. , … Bertolino, A. (2014). DRD2/CHRNA5 interaction on prefrontal biology and physiology during working memory. PLoS One, 9, e95997 10.1371/journal.pone.0095997 24819610 PMC4018353

[mgg3869-bib-0006] Dineley, K. T. , Westerman, M. , Bui, D. , Bell, K. , Ashe, K. H. , & Sweatt, J. D. (2001). Beta‐amyloid activates the mitogen‐activated protein kinase cascade via hippocampal alpha7 nicotinic acetylcholine receptors: In vitro and in vivo mechanisms related to Alzheimer's disease. Journal of Neuroscience, 21, 4125–4133. 11404397 10.1523/JNEUROSCI.21-12-04125.2001 PMC6762764

[mgg3869-bib-0007] Dupont, W. D. , & Plummer, W. D. Jr (1998). Power and sample size calculations for studies involving linear regression. Controlled Clinical Trials, 19, 589–601. 10.1016/S0197-2456(98)00037-3 9875838

[mgg3869-bib-0008] Fowler, C. D. , Lu, Q. , Johnson, P. M. , Marks, M. J. , & Kenny, P. J. (2011). Habenular α5 nicotinic receptor subunit signalling controls nicotine intake. Nature, 471, 597–601. 10.1038/nature09797 21278726 PMC3079537

[mgg3869-bib-0009] Freedman, R. , Adams, C. E. , & Leonard, S. (2000). The alpha7‐nicotinic acetylcholine receptor and the pathology of hippocampal interneurons in schizophrenia. Journal of Chemical Neuroanatomy, 20, 299–306. 11207427 10.1016/s0891-0618(00)00109-5

[mgg3869-bib-0010] Hansen, H. M. , Xiao, Y. , Rice, T. , Bracci, P. M. , Wrensch, M. R. , Sison, J. D. , … Wiencke, J. K. (2010). Fine mapping of chromosome 15q25.1 lung cancer susceptibility in African‐Americans. Human Molecular Genetics, 19, 3652–3661. 20587604 10.1093/hmg/ddq268 PMC2928127

[mgg3869-bib-0011] He, P. , Yang, X.‐X. , He, X.‐Q. , Chen, J. , Li, F.‐X. , Gu, X. , … He, J.‐X. (2014). CHRNA3 polymorphism modifies lung adenocarcinoma risk in the Chinese Han population. International Journal of Molecular Sciences, 15, 5446–5457. 10.3390/ijms15045446 24686516 PMC4013574

[mgg3869-bib-0012] Huang, C. Y. , Xun, X. J. , Wang, A. J. , Gao, Y. , Ma, J. Y. , Chen, Y. T. , … Gu, S. Z. (2015). CHRNA5 polymorphisms and risk of lung cancer in Chinese Han smokers. American Journal of Cancer Research, 5, 3241–3248. 26693074 PMC4656745

[mgg3869-bib-0013] Hung, R. J. , McKay, J. D. , Gaborieau, V. , Boffetta, P. , Hashibe, M. , Zaridze, D. , … Brennan, P. (2008). A susceptibility locus for lung cancer maps to nicotinic acetylcholine receptor subunit genes on 15q25. Nature, 452, 633–637. 10.1038/nature06885 18385738

[mgg3869-bib-0014] Le Marchand, L. , Derby, K. S. , Murphy, S. E. , Hecht, S. S. , Hatsukami, D. , Carmella, S. G. , … Wang, H. (2008). Smokers with the CHRNA lung cancer‐associated variants are exposed to higher levels of nicotine equivalents and a carcinogenic tobacco‐specific nitrosamine. Cancer Research, 68, 9137–9140. 10.1158/0008-5472.CAN-08-2271 19010884 PMC2587068

[mgg3869-bib-0015] Leucht, S. , Burkard, T. , Henderson, J. , Maj, M. , & Sartorius, N. (2007). Physical illness and schizophrenia: A review of the literature. Acta Psychiatrica Scandinavica, 116, 317–333. 10.1111/j.1600-0447.2007.01095.x 17919153

[mgg3869-bib-0016] Leucht, S. , Cipriani, A. , Spineli, L. , Mavridis, D. , Örey, D. , Richter, F. , … Davis, J. M. (2013). Comparative efficacy and tolerability of 15 antipsychotic drugs in schizophrenia: A multiple‐treatments meta‐analysis. Lancet, 382, 951–962. 10.1016/S0140-6736(13)60733-3 23810019

[mgg3869-bib-0017] Leucht, S. , Corves, C. , Arbter, D. , Engel, R. R. , Li, C. , & Davis, J. M. (2009). Second‐generation versus first‐generation antipsychotic drugs for schizophrenia: A meta‐analysis. Lancet, 373, 31–41. 10.1016/S0140-6736(08)61764-X 19058842

[mgg3869-bib-0018] Li, Z. , Bao, S. , Xu, X. , Bao, Y. , & Zhang, Y. (2012). Polymorphisms of CHRNA5‐CHRNA3‐CHRNB4 gene cluster and NSCLC risk in Chinese population. Translational Oncology, 5, 448–452. 10.1593/tlo.12304 23397474 PMC3567724

[mgg3869-bib-0019] Morel, C. , Fattore, L. , Pons, S. , Hay, Y. A. , Marti, F. , Lambolez, B. , … Faure, P. (2014). Nicotine consumption is regulated by a human polymorphism in dopamine neurons. Molecular Psychiatry, 19, 930–936. 10.1038/mp.2013.158 24296975 PMC8596967

[mgg3869-bib-0020] Moyer, R. A. , Wang, D. , Papp, A. C. , Smith, R. M. , Duque, L. , Mash, D. C. , & Sadee, W. (2011). Intronic polymorphisms affecting alternative splicing of human dopamine D2 receptor are associated with cocaine abuse. Neuropsychopharmacology, 36, 753–762. 10.1038/npp.2010.208 21150907 PMC3055737

[mgg3869-bib-0021] Niu, X. , Chen, Z. , Shen, S. , Liu, Y. , Zhou, D. , Zhang, J. , … He, L. (2010). Association of the CHRNA3 locus with lung cancer risk and prognosis in Chinese Han population. Journal of Thoracic Oncology, 5, 658–666. 10.1097/JTO.0b013e3181d5e447 20234319

[mgg3869-bib-0022] Shen, B. , Zhu, Q. , Zheng, M. Q. , Chen, J. , Shi, M. Q. , & Feng, J. F. (2013). CHRNA5 polymorphism and susceptibility to lung cancer in a Chinese population. Brazilian Journal of Medical and Biological Research, 46, 79–84. 10.1590/1414-431X20122451 23314339 PMC3854344

[mgg3869-bib-0023] Thorgeirsson, T. E. , Geller, F. , Sulem, P. , Rafnar, T. , Wiste, A. , Magnusson, K. P. , … Stefansson, K. (2008). A variant associated with nicotine dependence, lung cancer and peripheral arterial disease. Nature, 452, 638–642. 10.1038/nature06846 18385739 PMC4539558

[mgg3869-bib-0024] Uher, R. (2014). Gene‐environment interactions in severe mental illness. Frontiers in Psychiatry, 5, 48. 24860514 10.3389/fpsyt.2014.00048 PMC4030208

[mgg3869-bib-0025] Xie, A. , Gao, J. , Xu, L. , & Meng, D. (2014). Shared mechanisms of neurodegeneration in Alzheimer's disease and Parkinson's disease. BioMed Research International, 2014, 648740 10.1155/2014/648740 24900975 PMC4037122

[mgg3869-bib-0026] Xu, Z. W. , Wang, G. N. , Dong, Z. Z. , Li, T. H. , Cao, C. , & Jin, Y. H. (2015). CHRNA5 rs16969968 polymorphism association with risk of lung cancer‐evidence from 17,962 lung cancer cases and 77,216 control subjects. Asian Pacific Journal of Cancer Prevention, 16, 6685–6690. 10.7314/APJCP.2015.16.15.6685 26434895

[mgg3869-bib-0027] Zhou, W. , Geng, T. , Wang, H. , Xun, X. , Feng, T. , Zou, H. , … Chen, C. (2015). CHRNA3 genetic polymorphism and the risk of lung cancer in the Chinese Han smoking population. Tumour Biology, 36, 4987–4992. 10.1007/s13277-015-3149-0 25656608

