# Supplementary material for: Impact of CHRNA5 polymorphisms on the risk of schizophrenia in the Chinese Han population
Source: Mol Genet Genomic Med. 2019 Jul 24;7(9):e869. doi: 10.1002/mgg3.869 (PMC6732284; doi:10.1002/mgg3.869)
Supplement: Supplementary file 1 [file MGG3-7-e869-s001.docx]

Supplementary Table S1. The expression of *CHRNA5* SNPs in brain tissues

| SNP |  | Effect Size | *p*-value |  | Tissue |
| --- | --- | --- | --- | --- | --- |
|  |  |  |  |  |  |
| rs17486278 |  | -0.65 | 7.8 x 10^−8^ |  | Brain - Cortex |
| rs17486278 |  | -0.62 | 5.7 x 10^−7^ |  | Brain - Nucleus accumbens (basal ganglia) |
| rs17486278 |  | -0.59 | 1.4 x 10^−6^ |  | Brain - Caudate (basal ganglia) |
| rs17486278 |  | -0.63 | 3.8 x 10^−6^ |  | Brain - Substantia nigra |
| rs17486278 |  | -0.61 | 5.1 x 10^−6^ |  | Brain - Frontal Cortex (BA9) |
| rs588765 |  | -0.76 | 6.1 x 10^−18^ |  | Brain - Nucleus accumbens (basal ganglia) |
| rs588765 |  | -0.79 | 2.0 x 10^−17^ |  | Brain - Frontal Cortex (BA9) |
| rs588765 |  | -0.88 | 4.6 x 10^−17^ |  | Brain - Cortex |
| rs588765 |  | -0.85 | 1.5 x 10^−15^ |  | Brain - Anterior cingulate cortex (BA24) |
| rs588765 |  | -0.80 | 3.6 x 10^−15^ |  | Brain - Putamen (basal ganglia) |
| rs588765 |  | -0.72 | 1.3 x 10^−14^ |  | Brain - Caudate (basal ganglia) |
| rs588765 |  | -0.75 | 5.1 x 10^−14^ |  | Brain - Hypothalamus |
| rs588765 |  | -0.71 | 4.2 x 10^−12^ |  | Brain - Hippocampus |
| rs588765 |  | -0.67 | 8.6 x 10^−9^ |  | Brain - Substantia nigra |
| rs588765 |  | -0.49 | 6.9 x 10^−8^ |  | Brain - Cerebellar Hemisphere |
| rs588765 |  | -0.64 | 9.3 x 10^−8^ |  | Brain - Amygdala |
| rs588765 |  | -0.46 | 1.3 x 10^−7^ |  | Brain - Cerebellum |
| rs6495306 |  | -0.76 | 6.1 x 10^−18^ |  | Brain - Nucleus accumbens (basal ganglia) |
| rs6495306 |  | -0.79 | 2.0 x 10^−17^ |  | Brain - Frontal Cortex (BA9) |
| rs6495306 |  | -0.88 | 4.6 x 10^−17^ |  | Brain - Cortex |
| rs6495306 |  | -0.85 | 1.5 x 10^−15^ |  | Brain - Anterior cingulate cortex (BA24) |
| rs6495306 |  | -0.80 | 3.6 x 10^−15^ |  | Brain - Putamen (basal ganglia) |
| rs6495306 |  | -0.72 | 1.3 x 10^−14^ |  | Brain - Caudate (basal ganglia) |
| rs6495306 |  | -0.75 | 5.1 x 10^−14^ |  | Brain - Hypothalamus |
| rs6495306 |  | -0.71 | 4.2 x 10^−12^ |  | Brain - Hippocampus |
| rs6495306 |  | -0.67 | 8.6 x 10^−9^ |  | Brain - Substantia nigra |
| rs6495306 |  | -0.49 | 6.9 x 10^−8^ |  | Brain - Cerebellar Hemisphere |
| rs6495306 |  | -0.64 | 9.3 x 10^−8^ |  | Brain - Amygdala |
| rs6495306 |  | -0.46 | 1.3 x 10^−7^ |  | Brain - Cerebellum |
| rs680244 |  | -0.77 | 1.6 x 10^−16^ |  | Brain - Frontal Cortex (BA9) |
| rs680244 |  | -0.73 | 1.9 x 10^−16^ |  | Brain - Nucleus accumbens (basal ganglia) |
| rs680244 |  | -0.84 | 1.2 x 10^−15^ |  | Brain - Cortex |
| rs680244 |  | -0.85 | 1.5 x 10^−15^ |  | Brain - Anterior cingulate cortex (BA24) |
| rs680244 |  | -0.78 | 1.4 x 10^−14^ |  | Brain - Putamen (basal ganglia) |
| rs680244 |  | -0.72 | 4.2 x 10^−13^ |  | Brain - Hypothalamus |
| rs680244 |  | -0.68 | 7.4 x 10^−13^ |  | Brain - Caudate (basal ganglia) |
| rs680244 |  | -0.66 | 1.0 x 10^−10^ |  | Brain - Hippocampus |
| rs680244 |  | -0.64 | 1.3 x 10^−8^ |  | Brain - Substantia nigra |
| rs680244 |  | -0.64 | 9.3 x 10^−8^ |  | Brain - Amygdala |
| rs680244 |  | -0.44 | 3.3 x 10^−7^ |  | Brain - Cerebellum |
| rs680244 |  | -0.45 | 8.6 x 10^−7^ |  | Brain - Cerebellar Hemisphere |
| rs692780 |  | -0.96 | 1.1 x 10^-15^ |  | Brain - Cortex |
| rs692780 |  | -0.80 | 8.8 x 10^-15^ |  | [Brain - Frontal Cortex (BA9)](javascript:portalClient.eqtl.goTissuePage('Brain_Frontal_Cortex_BA9')) |
| rs692780 |  | -0.75 | 3.9 x 10^-14^ |  | [Brain - Caudate (basal ganglia)](javascript:portalClient.eqtl.goTissuePage('Brain_Caudate_basal_ganglia')) |
| rs692780 |  | -0.69 | 4.3 x 10^-13^ |  | [Brain - Nucleus accumbens (basal ganglia)](javascript:portalClient.eqtl.goTissuePage('Brain_Nucleus_accumbens_basal_ganglia')) |
| rs692780 |  | -0.79 | 1.7 x 10^-12^ |  | Brain - Putamen (basal ganglia) |
| rs692780 |  | -0.78 | 1.4 x 10^-11^ |  | [Brain - Anterior cingulate cortex (BA24)](javascript:portalClient.eqtl.goTissuePage('Brain_Anterior_cingulate_cortex_BA24')) |
| rs692780 |  | -0.73 | 2.0 x 10^-10^ |  | [Brain - Hippocampus](javascript:portalClient.eqtl.goTissuePage('Brain_Hippocampus')) |
| rs692780 |  | -0.67 | 6.9 x 10^-9^ |  | [Brain - Hypothalamus](javascript:portalClient.eqtl.goTissuePage('Brain_Hypothalamus')) |
| rs692780 |  | -0.64 | 5.2 x 10^-7^ |  | [Brain - Substantia nigra](javascript:portalClient.eqtl.goTissuePage('Brain_Substantia_nigra')) |
| rs692780 |  | -0.47 | 9.8 x 10^-7^ |  | [Brain - Cerebellum](javascript:portalClient.eqtl.goTissuePage('Brain_Cerebellum')) |
| rs692780 |  | -0.43 | 1.9 x 10^-5^ |  | [Brain - Cerebellar Hemisphere](javascript:portalClient.eqtl.goTissuePage('Brain_Cerebellar_Hemisphere')) |
